# Management of scrotal pruritus with topical roflumilast 0.3% once daily treatment

**DOI:** 10.1016/j.jdcr.2024.03.002

**Published:** 2024-03-08

**Authors:** Paul S. Yamauchi

**Affiliations:** Division of Dermatology, David Geffen School of Medicine, University of California, Los Angeles, Los Angeles, California

**Keywords:** PDE4 treatment for scrotal pruritus, pruritus treatment, roflumilast, roflumilast 0.3%, scrotal pruritus management, scrotal pruritus treated with topical roflumilast 0.3%

## Introduction

Many inflammatory skin diseases such as atopic dermatitis, psoriasis, and seborrheic dermatitis can cause pruritus. Pruritus is characterized by an excessive desire to scratch or “itch” the unpleasant feeling on the skin and can be characterized as either acute or chronic.^1^ Scratching an itch can be intensely desirable or intensely bothersome if done repeatedly, especially when the pruritus is chronic (lasting >6 weeks) which affects 15% of the population.^1^ Itch is rarely a pure sensation, but instead, typically includes degrees of burning, prickling, and stinging.^1^ The pathophysiology of itch is complex and includes both endogenous (neurologic and immune involvement) and exogenous (allergens, pruritogens, and irritants) that interact to ultimately lead to the itch-scratch cycle.^1^ In this case series, we report 3 different cases of patients experiencing scrotal pruritus that were refractory to topical therapies including steroids, calcineurin inhibitors, and Janus kinase (JAK) inhibitors. In these cases, pruritus affected sleep quality and impacted quality of life. Roflumilast cream 0.3% is a highly selective, nonsteroidal and potent topical phosphodiesterase 4 inhibitor approved in 2022 by the Food and Drug Administration for the treatment of psoriasis, including intertriginous disease. In these 3 patients, effective management of itch was achieved with roflumilast 0.3% once daily therapy.

## Case 1

Here is a case of 39-year-old man with a 6-month history of intense pruritus of the scrotum. The patient described his pruritus as disturbing his sleep and thus affecting his quality of life. The patient’s prior therapy included intermittent courses of mid and high potency topical steroids for 2 weeks. The patient stated that these provided relief, but the pruritus returned after a few days after discontinuation. Additionally, antihistamines and topical calcineurin inhibitors demonstrated minimal relief. The patient had an unremarkable past medical history and no family history of psoriasis. The patient, an attorney, is a social drinker and nonsmoker. Upon presentation, the scrotum had moderate erythema and accentuated skin markings. Triamcinolone 0.1% cream was discontinued and roflumilast 0.3% cream once daily was prescribed which resulted in maintained pruritus relief and after 1 month, the erythema had subsided. Upon roflumilast 0.3% discontinuation, the pruritus relapsed after 2 weeks but resolved when treatment was subsequently restarted.

## Case 2

Here is the case of a 45-year-old man with a 3-month history of intense pruritus of the scrotum. The patient reported sleep disturbances and embarrassment from itching that impacted his quality of life. The patient’s prior therapies included moderate potency topical corticosteroids, topical calcineurin inhibitors and a topical JAK inhibitor. Itch resolution, however, was only achieved with topical steroids. The patient’s past medical history was unremarkable only having hypertension. There was no family history of psoriasis. The patient reported being a social drinker, nonsmoker, and works as a real estate agent. Upon presentation, the scrotum was erythematous with lichenification. Roflumilast 0.3% cream once daily monotherapy was initiated and the patient experienced itch improvement after 3 days. Application was continued daily and at the 6-week follow-up there was no evidence of erythema. The patient continued to apply roflumilast 0.3% as needed.

## Case 3

Here is the case of a 41-year-old man with a 2-year history of intense scrotal pruritus. The patient reported a decreased quality of life with sleep disturbances and depression from his chronic pruritus. Prior therapy included mid to high potency topical steroids, topical calcineurin inhibitors, a topical JAK inhibitor, gabapentin 300 mg 3 times a day, pregabalin 75 mg twice a day, naltrexone 50 mg every day, and antihistamines. The patient had attempted oral apremilast 30 mg twice a day but discontinued due to diarrhea and headaches. Only high potency steroids provided pruritus relief. The patient had an unremarkable past medical history and does not have a family history of psoriasis. The patient reported being a social drinker, nonsmoker, and works as an oral surgeon. Upon presentation, the scrotum was erythematous with severe lichenification. Roflumilast 0.3% was prescribed to use nightly along with clobetasol 0.05% cream daily in the morning. Once pruritus resolved, the clobetasol 0.05% was discontinued and roflumilast 0.3% once daily was continued as monotherapy. Resolution continued with roflumilast 0.3% alone and at 6-week follow-up there was decrease in lichenification of the scrotum and minimal erythema. The patient continued to use roflumilast 0.3% as maintenance therapy daily with triamcinolone 0.1% cream applied sparingly as needed for relapse of the pruritus.

## Discussion

These case reports of 3 adult men, all suffering from intense scrotal pruritus, serve to highlight the magnitude of effect that pruritus can evoke in someone’s life. In all 3 cases, patients reported impact on their quality of life due to embarrassment, depression, and reduced sleep. Additionally, paradigm treatments such as topical corticosteroids, topical calcineurin inhibitors, JAK inhibitors all had suboptimal efficacy in these patients. Medium and high potency steroids are effective at resolving pruritus but are limited by their long-term safety profile and complex dosing schedules. Daily treatment with roflumilast 0.3% cream alongside topical corticosteroids resulted in resolution of itch and a maintained response even after discontinuation of the topical corticosteroids. These cases additionally serve to highlight the unmet need of patients experiencing chronic pruritus and serve as evidence that roflumilast 0.3% cream, approved for the treatment of psoriasis, may be a safe and novel treatment that can increase patient outcomes and enhance their quality of life. Future clinical evaluation is indicated to further evaluate the role that topical roflumilast 0.3% can have on scrotal pruritus.

## Conflicts of interest

Dr Yamauchi reported honoraria from Arcutis Biotherapeutics during the conduct of the case study and honoraria from Amgen, AbbVie, Lilly, Janssen, UCB, LEO Pharma, Dermavant, Bristol Myers Squibb, Novartis, and Sun Pharma outside the submitted work.
